# Histological and Immunohistochemical Characterization of Vascular Alterations in Meninges of Cats Infected with *Gurltia paralysans*

**DOI:** 10.3390/pathogens11010088

**Published:** 2022-01-12

**Authors:** Svenja Hartung, Angelika Weyrich, Manuel Moroni, Marcelo Gómez, Christiane Herden

**Affiliations:** 1 Department of Veterinary Medicine, Institute of Veterinary-Pathology, Justus-Liebig-University Giessen, 35392 Giessen, Germany; Christiane.Herden@vetmed.uni-giessen.de; 2 Instituto de Patología Animal, Facultad de Ciencias Veterinarias, Universidad Austral de Chile, Valdivia 5090000, Chile; manuelmoroni@uach.cl; 3 Instituto de Farmacología y Morfofisiología, Facultad de Ciencias Veterinarias, Universidad Austral de Chile, Valdivia 5090000, Chile; marcelogomez@uach.cl

**Keywords:** *Gurltia paralysans*, gurltiosis, parasitic paraparesis, emerging disease, felines

## Abstract

*Gurltia paralysans*, a metastrongyloid nematode, parasitizes in meningeal vessels in the thoracolumbar spinal cord of cats in South America and causes progressive paraparesis. Recently, the first report outside of South America described gurltiosis in a cat in Spain. As this parasitic disease has so far been largely neglected, especially outside of South America, the aim of the present case study was to add knowledge to the histologic and immunohistochemical characterization of central nervous lesions. To this purpose, formalin-fixed and paraffin-embedded (FFPE) tissue samples from the spinal cord and brain of five cats affected by clinical signs caused by *Gurltia paralysans* and of three control cats without CNS lesions were histopathologically examined using hematoxylin and eosin stain (HE), Elastica van Gieson stain, as well as periodic acid–Schiff (PAS) reaction. Moreover, immuno- histochemistry for alpha smooth muscle actin and Factor VIII-related antigen were performed to characterize vascular lesions. Lesions were consistent with previous descriptions and were mainly located in the spinal cord and consisted of chronic suppurative or lymphoplasmahistiocytic meningi tis as well as suppurative vasculitis, congestion and varicosis of meningeal veins. In view of the recent detection of this parasite in Europe and the increasing inner-European transport of rescued domestic cats, veterinarians in Europe should be aware of the clinical and pathomorphological presentation of this disease.

## 1. Introduction

*Gurltia paralysans* is a nematode of the family Angiostrongylidae which is known to parasitize in the meningeal vessels of wild ranging and domestic felids, causing clinical disease dominated by progressive paraparesis and paraplegia and was first described in 1933 by Kurt Wolfgang Wolffhügel [1]. There are few case reports from Southern America, including cases in southern Chile (especially Región de los Lagos, Región de los Ríos, Región de la Araucania) [2,3], Brazil [4,5], Uruguay [6], Colombia and Argentina [7]. Recently, there has been one case description from Tenerife, Spain, an island located at the Moroccan coast [8]. Clinical disease is known in domestic felids, as well as in free-ranging northern tiger cats (*Leopardus tigrinus*) and margay (*Leopardus wiedii*) in Brazil [3,4]. Kodkods (*Leopardus guigna*) in Chile [9] and Geoffroy’s cat (*Leopardus geoffroyi*) [10] are thought to be the natural definitive hosts, but clinical disease has not yet been described in these species [7]. The life cycle is not known so far, but gastropods are proposed to be the intermediate host [2]. Sepulveda-Garcia et al. failed in demonstrating *Gurltia* sp. in various gastropods from southern Chile [11]. Disease in domestic cats is dominated by paraparesis, paraplegia, hindlimb ataxia, hindlimb proprioceptive deficit, urinary incontinence, decrease superficial and/or deep pain sensation in the hindlimbs, tail and anal atony with a slow clinical course of many months to two years [2,3,5,6,7]. Pathological changes include acute meningeal congestion and hemorrhages [2,3,7]. The described histopathological changes are non-suppurative meningitis [6,7], thrombosis of the meningeal vessels, vessel dilation and extensive tortuous vessels [2,5,7]. In all cases, adult nematodes and eggs are histologically detected in subarachnoid blood vessels [2,3,6]. Myelodegenerative disease with axon degeneration and gliosis due to spinal cord compression is common [2,3,6]. In one case, ocular gurltiosis was described. This case also marked the first description of gurltiosis outside of South America in Europe (Isle of Tenerife, Spain) [8]. Herein lies one major aspect making this disease and its pathomorphological and clinical changes relevant to veterinary pathologists and practitioners in Europe and around the world. The aim of the present case study was to add to the pathomorphological descriptions of this neglected parasitosis by histological and immunohistochemical characterization of central nervous tissue lesions.

## 2. Results

The examined material included the cerebellum and brain stem, different regions of the spinal cord, and cerebrum with or without the hippocampus. In all animals from southern Chile, an infection with *G. paralysans* had been diagnosed during necropsy by the presence of adult, female and male nematodes in the leptomeningeal vessels. All affected animals had shown signs of progressive myelopathy before their death (see Table 1 for further information on cats).

**Figure 1:**
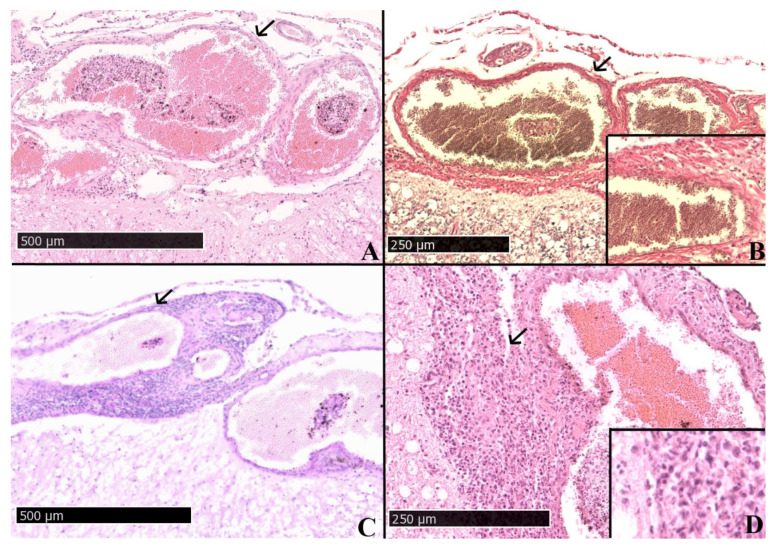
Adult domestic feline. Animal No. 4 (398-14-N). (**A**) Spinal cord, multiple dilated and congested meningeal veins (arrow), HE, 8×, bar = 500 µm (**B**) Spinal cord, dilated and congested meningeal veins (arrow), Elastica van Gieson, 15×, bar = 250 µm; Inset: Elastica van Gieson, 10×; moderate perivenous fibrosis (**C**) Spinal cord, dilated and congested meningeal veins (arrow), PAS reaction, 8×, bar = 500 µm (**D**) Spinal cord, chronic suppurative meningitis (arrow), HE, 15×, bar = 250 µm; Inset: inflammatory infiltrate, HE, 30×.

Histological examination of the spinal cord of two animals revealed dilation and acute congestion of the meningeal veins in the spinal cord (Figure 1A–C). Multiple cross sections of tortuously arranged dilated veins were detected. Furthermore, there was a moderate perivenous accumulation of fibrous connective tissue in the spinal cord of animals No. 3 and 4, indicative of perivenous fibrosis (Figure 1B). Perivenous fibrosis could be highlighted with Elastica van Gieson staining, in which fibrous connective tissue is colored intensely red. In both animals, inflammatory changes were found: chronic suppurative meningitis (animal No. 4, Figure 1D) and lymphoplasmahistiocytic meningitis (Animal 3), respectively. In animal No. 4, neutrophiles were also found in vessel walls consistent with vasculitis or exocytosis due to meningitis (Figure 1D). Dilated myelin sheaths were present in the spinal nerves of animal No. 4. 

In the cerebrum, cerebellum and brain stem, the meningeal vessels were congested and mild lymphoplasmahistiocytic meningeal infiltrates were found. Other vascular lesions (especially perivascular fibrosis) were not detected in the tissue samples from the brains of five affected cats. One animal (No. 1) had chronic suppurative meningitis in the brain, especially in the hippocampus. Animal 2 showed spheroid formation in the brain stem.

None of the available tissue sections of the five affected cats contained eggs, larvae or adult nematodes. Additionally, when applying the PAS reaction for highlighting affected areas, no parasites were detected.

**Figure 2:**
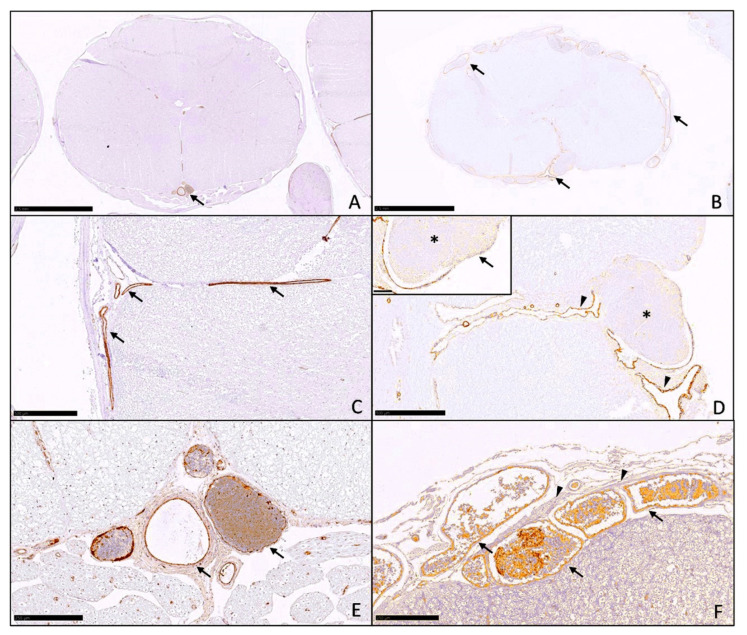
Immunohistochemistry. (**A**) Control animal No. 7: Spinal cord, markedly fewer cross sections of veins (arrow) compared to (**B**) no dilation and minimal congestion, SMA, 12×, bar 2.5 mm B. Animal No.4: Spinal cord, multiple dilated and congested meningeal veins (arrows), SMA, 12×, bar 2.5 mm (**C**) Control animal No. 7: Spinal cord, small arteries and vein without histopathological changes, continuous vessel wall (arrows), SMA, 50×, bar 500 μm (**D**) Animal No. 4: Spinal cord, dilated and tortuous meningeal veins (arrowheads), fibrin thrombus (asterisk) obliterating the vessel lumen, SMA, 50×, bar 500 μm; Inset: Thrombophlebitis, fibrin thrombus (asterisk) obliterating the vessel lumen, fibrin and inflammatory cells obscuring and infiltrating the vessel wall (arrow), SMA, 200×, bar 100 μm (**E**) Control animal No. 7: Spinal cord, no tortuous veins but only single round cross sections of meningeal vessels (arrows), Factor VIII, 100×, bar 250 μm (**F**) Animal No. 4: Spinal cord, dilated, congested and tortuous meningeal veins (arrows) with surrounding fibrosis (arrowheads), Factor VIII, 100×, bar 250 μm; images have been digitally edited to reduce background staining.

Immunohistochemistry helped in the visualization of vascular alterations. Alpha smooth muscle actin (SMA) was especially useful in demonstrating the difference between infected (Figure 2A,C) and uninfected (Figure 2B,D) cats. Remarkable venous dilation and congestion (in all infected animals), as well as thrombophlebitis with infiltration and discontinuation of vessel walls (in animal No. 4, Figure 2C) was evident. Immunohistochemistry was particularly useful to facilitate retracing of the vessel walls in view of advanced autolytic changes of the tissue samples. Control animals showed fewer meningeal vessels with round cross sections (Figure 2F), whereas infected cats all displayed multiple closely adjacent, irregularly shaped cross sections of tortuous varicose veins (Figure 2E). These were mainly detectable in the leptomeninges of the spinal cord. In more cranial locations (brain stem, cerebellum and cerebrum), no differences were visible between infected cats and the control group.

## 3. Discussion

The pathohistological findings in the presented cases were largely consistent with previously described lesions [2,3,4,5,7,12,13]. They mainly encompassed the leptomeninges of the spinal cord and consisted of chronic suppurative or lymphoplasmahistiocytic meningitis as well as suppurative thrombophlebitis of meningeal veins. Severe congestion and tortuous arrangement of varicose veins could be demonstrated. Meningeal hemorrhages were largely absent, and major changes in the neuropil of the spinal cord were also not visible. Solely animal No. 4 had mild dilation of myelin sheaths in spinal nerves. Apart from one animal (No. 1), the inflammatory cell infiltrates in the brain areas investigated and the brainstem were only mild. Furthermore, vascular changes, except of acute congestion, were not detected in the brain. In contrast to our cases, previous case descriptions reported myelodegenerative disease with axon degeneration and gliosis, panmyelomalacia as well as inflammatory infiltrates in the spinal cord parenchyma and extensive hemorrhage in proximity to parasites [2,3,12,13]. In this context, the absence of parasitic stages in our tissue samples could be relevant. This could be due to the advanced stage of disease. Likely, damage to spinal cord tissue is more pronounced in close proximity to adult nematodes or large numbers of eggs, since obliteration of vessels and subsequent varicosity and blood stasis leads to compression of spinal cord parenchyma and nerve roots. This is assumed to play a major role in the pathogenesis of spinal cord lesions [2,13]. Additionally, parasitic migration inside of blood vessels and into the spinal cord parenchyma might cause direct tissue damage and local inflammatory reactions [2]. Generally, venous congestion and dilation was milder in rostral parts of the central nervous system, similar to the observations published [14], but might also have been caused by agonal congestion. The distribution of lesions is consistent with the main localization of adult nematodes in subarachnoid vessels of the lumbar spinal cord [2,3,5,6,7,12,13]. However, the extent of venous congestion, varicosis and thrombophlebitis was remarkable, even in the absence of nematode stages in the affected tissue sections. These changes, together with the perivenous fibrosis, support evidence of a chronic disease stage. Further investigation will be necessary to elucidate the pathogenic mechanisms that promote thrombophlebitis and other vascular changes. Our findings support the theory that other factors apart from turbulent blood flow and blood stasis via mechanical obstruction of veins promote thrombosis. The possible effect of *G. paralysans* on the release of inflammatory mediators and prothrombotic cytokines is worth investigation.

Cats infected with *G. paralysans* in South America are often reported to have coinfection with *Aelurostrongylus abstrusus* [7,12,15,16]. *A. abstrusus* infection is common in cats in Europe [17,18] and causes vascular lesions which consist of endothelial swelling and proliferation as well as widespread medial hypertrophy and arteritis due to larval migration [19]. Intermediate hosts for *A. abstrusus* are different species of snails and slugs [18,20,21]. Birds, amphibians and reptiles serve as paratenic hosts [17]. Since the intermediate host of *G. paralysans* is not known, a similarity to those of *A. abstrusus* must be taken into consideration, especially in view of coinfections in cats with *G. paralysans*. Due to climate change, the habitats of possible intermediate hosts can change, and might expand to the northern hemisphere.

Intra vitam diagnosis of *G. paralysans* is currently under investigation but has to be further validated for routine use [12,15]. Until this are widely available, it will not be possible to test cats before import from South America to Europe or before inner-European transport. In view of the substantial and increased travel of pet animals with their owners, or due to the importation of rescued feral cats from the Mediterranean, veterinary practitioners and pathologists in Europe should be aware of feline gurltiosis and its clinical and pathomorphological presentation [8]. In the context of climate change, increasing transports of housed animals and animal transports, which are especially performed by animal welfare organizations in Europe, gurltiosis must be considered as a rare but possible differential diagnosis as a potential new emerging disease in cats. Tissue samples from the lumbar and sacral spinal cord should be collected for histological examination in suspected cases of gurltiosis. The presented report contributes to the number of case studies available and hopefully helps to increase recognition of this neglected parasite.

## 4. Material and Methods

Formalin-fixed and paraffin-embedded (FFPE) tissue specimens of the CNS of five domestic cats from southern Chile and of three control cats (No. 6–8) from routine post-mortem diagnostics of the Institute of Veterinary Pathology (Giessen, Germany) without central nervous lesions were examined histologically and immunohistochemically. All FFPE tissue samples were sectioned at 4 µm and routinely stained with hematoxylin and eosin stain (HE). Elastica van Gieson stain and a periodic acid-Schiff (PAS) reaction were performed to demonstrate blood vessel architecture and to visualize parasites, respectively. Furthermore, immunohistochemistry was performed on all sections using a two-day protocol with incubation of primary antibodies against alpha smooth muscle actin (SMA) and Factor VIII-related antigens at 4 °C overnight and utilizing 3,3’-diaminobenzidine as chromogen (see Table 2 for antibodies and detection systems used).

## Figures and Tables

**Table 1:** Origin, age, gender, and clinical presentation and sampled anatomical regions of the examined animals sampled. No. 6–8 are control animals without G. paralysans infection.

Animal Number	Age	Gender	Origin	Clinical Presentation	Sample Regions in CNS
1	10 years	Male	Punucapa, Valdi- via, Los Rios region (Southern Chile)	Paraplegia, loss of superficial and deep pain in pelvic limbs, decrease in tail and anal tone	Cerebrum, cerebellum, brain stem
2	2 years	Female	Puerto Varas, Los Lagos region (Southern Chile)	Paraplegia, decrease of superficial pain in pelvic limbs, in of tail tone	Hippocampus, cerebellum, brain stem
3	2 years	Female	Punucapa, Valdi- via, Los Ríos re- gion (Southern Chile)	Pelvic limb ataxia, ambulatory para- paresis	Cerebrum, cerebellum, brain stem, spinal cord with spinal nerves
4	3 years	Male	Paillaco, Itropulli area, Los Ríos region (Southern Chile)	Non-ambulatory paraparesis, loss of superficial and deep pain in pelvic limbs, decrease in tail and anal tone	Cerebrum with hippocampus, cere- bellum, brain stem, spinal cord with spinal nerves
5	10 years	Female	Paillaco, sector Itropulli, Los Ríos region (Southern Chile)	Paraplegia, loss of superficial and deep pain in pelvic limbs, decrease in tail and anal tone	Cerebrum with hippocampus, cerebellum, brain stem
6	2 months	Male	Germany	Diarrhea, dehydration	Cerebrum, cerebellum, brain stem, spinal cord
7	4 years	Female	Germany	Anorexia, sudden death	Cerebrum, cerebellum, spinal cord
8	18 years	Male	Germany	Paraparesis due to saddle thrombus in aortic bifurcation	Cerebrum with hippocampus, cerebellum, brain stem, spinal cord

**Table 2:** Antibodies and detection systems used for immunohistochemical examination.

Primary Antibody	Dilution	Blocking	Antigen Retrieval	Secondary Antibody	Detection System
alpha smooth muscle actin (SMA) (monoclonal mouse anti-human clone 1A4 ^1^)	1:500	Horse serum ^2^	Citrate pH 6	Biotinylated horse anti-mouse IgG ^3^	ABC ^3^
Factor VIII related antigen (polyclonal rabbit anti-human ^1^)	1:1000	20% pig serum ^2^	Protease	Pig anti-rabbit IgG ^3^	Rabbit PAP ^1^

Sources ^1^ DAKO Deutschland (Hamburg, Germany) ^2^ Thermo Fisher Scientific (Waltham, MA, USA) ^3^ Vector Laboratories (Burlingame, CA, USA) ABC: avidin-biotin complex method PAP: peroxidase anti-peroxidase method.

## Data Availability

Not applicable.
